# Clustering user preferences for personalized teleoperation control schemes via trajectory similarity analysis

**DOI:** 10.3389/frobt.2024.1330812

**Published:** 2024-04-09

**Authors:** Jennifer Molnar, Varun Agrawal, Sonia Chernova

**Affiliations:** ^1^ Woodruff School of Mechanical Engineering, Georgia Institute of Technology, Atlanta, GA, United States; ^2^ School of Interactive Computing, Georgia Institute of Technology, Atlanta, GA, United States

**Keywords:** HRI, human-robot interaction, user-centered design, virtual reality, VR, teleoperation, control schemes, remote-control

## Abstract

Successful operation of a teleoperated robot depends on a well-designed control scheme to translate human motion into robot motion; however, a single control scheme may not be suitable for all users. On the other hand, individual personalization of control schemes may be infeasible for designers to produce. In this paper, we present a method by which users may be classified into groups with mutually compatible control scheme preferences. Users are asked to demonstrate freehand motions to control a simulated robot in a virtual reality environment. Hand pose data is captured and compared with other users using SLAM trajectory similarity analysis techniques. The resulting pairwise trajectory error metrics are used to cluster participants based on their control motions, without foreknowledge of the number or types of control scheme preferences that may exist. The clusters identified for two different robots shows that a small number of clusters form stably for each case, each with its own control scheme paradigm. Survey data from participants validates that the clusters identified through this method correspond to the participants’ control scheme rationales, and also identify nuances in participant control scheme descriptions that may not be obvious to designers relying only on participant explanations of their preferences.

## 1 Introduction

Teleoperated robots, also called remote-controlled robots, exist to extend an operator’s reach or ability in situations where full robot autonomy is either not preferred or not possible. These robots are prominent in scenarios where environments are difficult or dangerous to access, such as in space (Tang et al., 2023), under water (Xu et al., 2021), in surgery (Li et al., 2023), and construction (Lee et al., 2022). Though teleoperation is most prominently used to extend an operator’s reach, it can also be used to extend their physical capabilities by allowing the user to take advantage of the robot’s specific embodiment (Toet et al., 2020). As an example, a human teleoperating an excavator is able to pick up large amounts of rubble with the excavator’s arm and bucket, a phenomenon that would be impossible with their human arm and hand (Gong et al., 2019). Similarly, a surgeon operating a laparoscopic surgical robot benefits not only from translating their motions to a different size and location, but can also choose end-effectors such as scissors, cautery tools, and needle drivers that offer more specific functions than comparatively hand-like options such as graspers (noa, 2023; Li et al., 2023).

To teleoperate a robot with a non-human morphology, a mapping must be designed to translate the operator’s inputs into the robot’s corresponding actions (Molnar and Mengüç, 2022). This mapping, called a “control scheme,” is constrained by the type of inputs that can be captured by the hardware that serves as the control interface. It also reflects a conceptual challenge known as the “Correspondence Problem” (Dautenhahn and Nehaniv, 2002), which can be paraphrased as: “Given two agents with different forms, how can the states, actions, or goals of one be mapped to the other?” Importantly, the correspondences between two agents may be perceived differently by different observers; thus, the best functional mapping between user and robot may be user-dependent.

The control scheme design challenge was reaffirmed as unsolved as recently as 2022 (Rea and Seo, 2022), in which paper the authors suggest that improvements from user-centered design have trailed improvements in technology, creating a situation in which the main factor limiting teleoperated robot usage is the difficulty of learning to effectively control them. A common approach to control scheme design is for a researcher to design several mappings and then compare user performance for each control scheme (Wang et al., 2018; Hetrick et al., 2020; Whitney et al., 2020; Meeker et al., 2022; Molnar and Mengüç, 2022). The control interface is usually pre-determined, and performance is task-dependent, so the researcher selects the performance evaluation tasks based on the particular use case for the robot (Molnar and Mengüç, 2022). In some studies with a more user-centered approach, the user may be asked to provide specific demonstrations using a provided control interface to indicate the control scheme they prefer (Li et al., 2020; Losey et al., 2020). Note that in all cases, the control interface exists as a constraining prior.

Recent improvements in Virtual Reality (VR) technology allow us to adopt a user-centered approach that works in the reverse order: we can ask users to demonstrate their preferred mappings, and then build a control interface to accommodate them. In this paper, we develop the tools necessary to capture information about the mappings users perceive between themselves and a virtual robot in VR. We then analyze this data for two sample robot arms to identify the control scheme paradigms demonstrated by each user. We then group users into categories based on these choices for two sample robot arms, and compare these groups with participants’ self-descriptions of their control scheme paradigms to validate a common control scheme concept for each group. To our knowledge, we are the first to examine the possibility of group-wise personalization of teleoperation control schemes, and to present a user-centered design method to objectively identify the existence and identity of such group-wise control scheme concepts.

## 2 Related work

Remotely-controlled moving machines as we know them began with Nikola Tesla near the turn of the 20th century, and has since progressed to encompass a wide variety of machines (Nof, 2009). Leveraging ever faster and more accessible communication methods, teleoperated robots continue to support human activity in remote, dangerous, or otherwise inaccessible environments (Nof, 2009). However, challenges still remain, including: the fidelity and latency of information transmission, presentation of that information to the operator in a useful way, the intuitive design of the control scheme and interface, and the level and functionality of the robot’s autonomy are all design features that are still being improved on (Lee et al., 2022). A survey of usability design guidelines for teleoperated robots mentions, among other factors, the need for the naturalness and efficiency of cues and the reduction of cognitive workload (Adamides et al., 2015). In fact, other researchers have posited that technological advances have outpaced improvements in user-experience design to the extent that a lack of trained and expert users is now the limiting factor in the adoption of teleoperated robotic systems, rather than any lack of those systems’ usefulness and functionality (Rea and Seo, 2022).

Virtual Reality has emerged as a promising teleoperation interface because it provides immersive, natural visual feedback that is intuitive to understand and produces a strong sense of presence (Rosen et al., 2018; Nostadt et al., 2020; Whitney et al., 2020). VR may also uniquely be able to create a sense of embodiment or ownership over the robot being controlled (Kilteni et al., 2012; Toet et al., 2020). Recent advances in virtual reality technology have made it possible to teleoperate robots from within a virtual environment in real time (Rosen et al., 2018; Whitney et al., 2018; Jang et al., 2019).

As VR-enabled teleoperation has become increasingly popular, it has also been leveraged to prototype teleoperation control schemes, allowing designers to test a variety of control schemes on test users without the time and cost overhead associated with mechanical prototyping (Devreker et al., 2016; Wonsick and Padir, 2020). Attention from the video game development community has also led to additional research on viable control schemes for teleoperating non-anthropomorphic avatars (Won et al., 2015; Krekhov et al., 2018). The native hand-tracking capabilities of certain VR headsets provide an additional valuable user interface, enabling direct hand-to-robot mapping as an alternative to a standard controller interface with joysticks and buttons (Zhang et al., 2018; Jang et al., 2019).

Controller interfaces are a critical component of a teleoperation system, as they define the information that can be gathered from the user’s movements. The control scheme that maps a user’s motion to the robot’s motion can only be designed based on the information that the control interface can provide. The most common control interfaces fall into three categories: video-game-style controllers with buttons and joysticks, master/puppet systems, and direct, human-as-the-master master/puppet systems (Gong et al., 2019; Li et al., 2019; Chen et al., 2023). These three kinds of systems each capture different kinds of information and are suitable for particular robots. The first, video-game-style controllers, are able to capture discrete degrees of freedom (DoF) in the form of button presses (binary data) and joysticks (continuous 1- or 2-DoF data). When the desired behavior of the robot can be described by individual degrees-of-freedom, this interface is highly useful and adaptable to many kinds of robots (Winck et al., 2015; de la Cruz et al., 2020). On the other hand, a master/puppet system is useful for creating an intuitive mapping between the local, master device and the remote puppet one (Glover et al., 2009; Park et al., 2021), although if there are any differences between the kinematics of the master and the puppet, a control scheme must be invented to translate between the two (Rebelo and Schiele, 2012). The final method, with the human-as-the-master, is similar to the master/puppet system but uses the human operator’s own body as the master device, tracking its movement via cameras, datagloves, or IMUs (Ha et al., 2015; Jang et al., 2019). In this case, control schemes must be invented to translate the operator’s motion into the joint space of the robot (Speeter, 1992; Rakita et al., 2017). This method of teleoperation is expected to be advantageous for the human operator, but its effectiveness is highly dependent on the construction of an adequate control scheme (Wang et al., 2021).

Evaluation of control scheme effectiveness is typically done by comparing user performance across a set of experimenter-designed control schemes on a predefined control interface (Rosa et al., 2015; El-Hussieny et al., 2018; Wang et al., 2018; Meeker and Ciocarlie, 2019). An alternative, more user-centered approach to control scheme design is to base the control scheme off of information acquired from users in the form of survey or motion data (Li et al., 2020; Losey et al., 2020; Bobu et al., 2023). Developing control schemes from user data is often an iterative process: in (Li et al., 2020; Losey et al., 2020), the researchers first solicited user motions in order to identify task-relevant latent features, which the authors of the second paper were able to use to program their robot. They then gathered a new round of user data in order to determine the mapping between user motions and the previously identified latent variables. Importantly, these control schemes were personalized to every user, and other papers support the premise that that some level of control scheme personalization is required in order to accommodate participants’ individual, internal models (Paleja and Gombolay, 2019; Schrum et al., 2022; Bobu et al., 2023).

## 3 Research questions

Our goal is to improve human-robot interaction by informing the design of future teleoperation control schemes, specifically for robotic embodiments that lack an obvious one-to-one mapping with the user and may be confusing to learn.

When designing control schemes for novel robots, designers can choose from a spectrum of personalization options, ranging from a single, universal control scheme on the one hand to completely individualized control schemes on the other. We hypothesize that neither extreme is required—that users vary in their control scheme preferences, but those preferences can be grouped into a finite set of schemes, suitable for development and deployment. In this paper, we present a method to identify some such clusters on two example robotic arms, one anthropomorphic and one non-anthropomorphic. Both arms have similar degrees of freedom, but different joint configurations, allowing us to examine the effect of anthropomorphic and non-anthropomorphic robot forms on user preferences.

**FIGURE 1:**
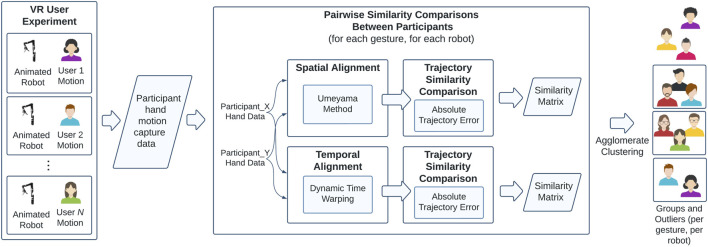
The design of the study. Users perform their own control motions to match virtual robot gestures. Their hand pose data (position and orientation) is captured by the VR headset. These user trajectories are then aligned spatially using Umeyama alignment and aligned temporally using Dynamic Time Warping (Umeyama, 1991; Salvador and Chan, 2007). Similarity between trajectories is computed using Absolute Trajectory Error (Grupp, 2017), which is used to cluster users into groups via agglomerative clustering. These groups of participants are those who invented similar control motions for particular gestures and robots, and thus can be collectively addressed for control scheme personalization.

In this user-centered design process, we asked participants to demonstrate the motions they would choose to use to control these two arms in a simulated, virtual environment. We developed a procedure (shown in Figure 1) that leverages trajectory analysis methods popularized by the SLAM community to objectively cluster users into groups based on the similarity of their control motions. We use the results of this analysis to answer the following questions:

1. If users are presented with a robot and offered the chance to design their own control scheme for it, do they naturally conform to a single control scheme, each invent their own personalized control scheme, or choose one of a small set of options?
2. Does robot embodiment or gesture type influence the kinds of control schemes that users invent?
3. Do users’ self-reported control schemes match the groupings identified by objective trajectory similarity analysis?

## 4 Methodology

In order to test the above questions, we developed a virtual reality (VR) study environment that simulates real robots (in the form of Unified Robotics Description Format files, or URDFs), which are pre-programmed using MoveIt (Robot Operating System software) (Sucan and Chitta, 2013; Unity Technologies, 2023). Users are able to observe the robot animations in 3D, and can record their own corresponding movements using the built-in hand-tracking functionality of the VR headset (Oculus Quest 2). This trajectory pose data is saved onto the headset in the form of CSV files. The similarity between participants’ control trajectories can then be calculated for each pair of participants and used as a basis for clustering to identify groups of participants with similar control scheme preferences.

Control motions for fifteen gestures were solicited for each robot, in the order listed in Table 1. Beginning with the Reachy robot, participants were instructed to play each gesture as many times as they desired. They were asked to imagine that the motion being observed was one what they wanted the robot to perform, and that they needed to convey this to the robot via their own movements. We were specifically interested in human-as-the-master master/puppet control schemes, so we informed the participants *a priori* that their hand and head poses were being recorded via the hand-held controllers, but any button presses and joystick movements they performed were not. The Oculus Quest two permits full hand-tracking, including finger movements; however, we constrained the scope of this study to controller-based, 6-DOF hand position and orientation tracking. Our simplification avoids possible errors in hand pose estimation that can occur when fingers overlap with each other from the headset camera’s perspective, while still capturing enough degrees-of-freedom from the human to form a complete mapping to the 6 DOF of the robot.

**TABLE 1:** Gesture selection for robots. Gestures were selected to include both free-form (G1-G6) and task-oriented motions (G7-G15). Task-oriented motions included ones that implied basic end-effector control (G7-G9), full-body control (G10-G12), continuous control (G13-G14), and one gesture with semantic rather than mechanical functionality (G15).

Gesture type	Gesture number	Reachy movement (right hand only)	Jaco movement
1 DoF Movements	1	Straight arm lift forward	Straight arm rotate right
	2	Straight arm lift right	Straight arm lift forward
	3	Rotate at shoulder	Mid-arm bend
	4	Elbow bend	Mid-arm twist
	5	Wrist twist	Distal bend
	6	Wrist bend	Distal twist
Touch a target	7	Reach up to target	Reach forward to target
	8	Reach right to target	Reach right to target
	9	Reach left to target	Reach left to target
Touch target around barrier	10	Reach underneath barrier to target	Reach over barrier to target
	11	Reach right around barrier to target	Reach left around barrier to target
	12	Reach over barrier on right to target	Reach over barrier on right to target
Push a block	13	Push block forward and to the left	Push block forward and to the right
	14	Push block backwards and to the right	Push block backwards and to the left
Communication	15	Wave	Wave (left side)

**FIGURE 2:**
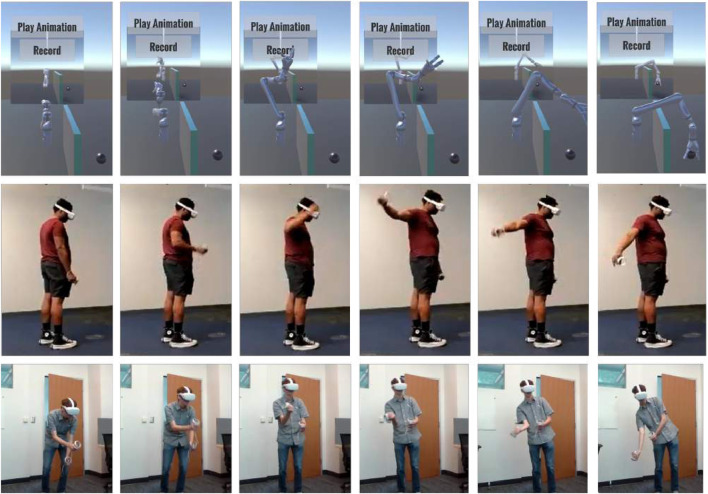
The Jaco robot performs a gesture (G10: “reach over barrier to touch a target”) in the VR test environment. Two GUI buttons allow the user to play the pre-defined animation, and to play it while recording their own gesture. A virtual mirror provides a reflected view of the robot for added visibility, although participants were permitted to walk around the robot in VR to observe it more closely if desired. Beneath the time-lapse image of the Jaco reaching over the virtual barrier are corresponding time lapses of two participants demonstrating the different motions they would like to use to control this gesture.

Once the participant had decided their command motion, they recorded five demonstrations of their command motion as the robot repeated its gesture (see Figure 2). The VR program prompted users for each demonstration and then prompted them to move on to the next gesture or robot. We video-recorded participants while they were in the VR app and asked to narrate their decision-making process in real time. The rationale behind their final control scheme, as well as any changes they made or would like to have made over the course of the study was recorded in a post-test survey. Additional background information for participants was also collected via survey, including details such as participant height, handedness, their participation in activities requiring fine or gross motor skills, and their familiarity with robots and VR. Our approach qualifies as a user-centered study, as we gather both observations from and by users in pursuit of a teleoperation control scheme design that meets their needs (Frascara, 2002).

### 4.1 Robot selection

**FIGURE 3:**
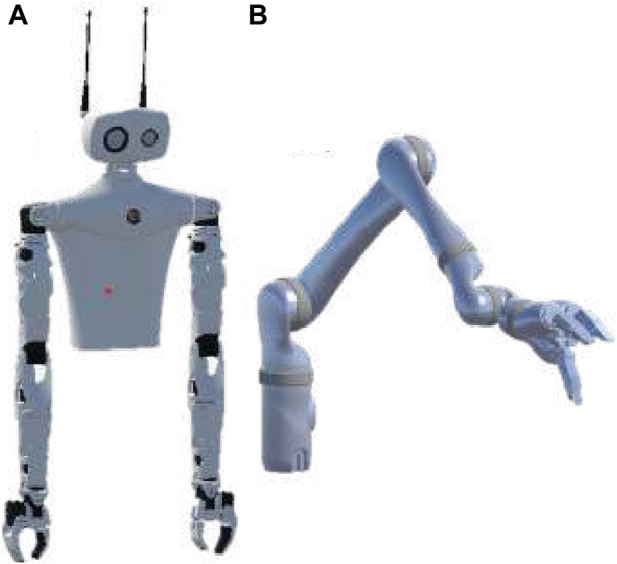
The two robots chosen as the targets for control scheme design. **(A)** the Reachy (Pollen Robotics Inc) is the baseline robot, with human-like dimensions and range of motion. Only its right arm is moved during the study. **(B)** a 6-DoF Jaco arm (Kinova Robotics Inc.) was the second robot participants were asked to design control movements for. Both robot arms have 6 DoF—matching the 6-DoF position and orientation data being recorded for each hand during the study.

The specific robots chosen for this study are shown in Figure 3: one anthropomorphic, the other non-anthropomorphic. The right arm of Pollen Robotics’ Reachy robot has a human-like configuration (Mick et al., 2019), providing a straightforward one-to-one structural mapping from user to robot if the participants so choose. The second robot, the Jaco arm from Kinova Robotics, has the same six degrees of freedom, but distributed differently throughout its non-anthropomorphic arm, rendering a one-to-one structural mapping impossible. This requires participants to invent alternative control schemes, which we can group by similarity to investigate Research Question 1 (RQ1).

### 4.2 Gesture selection

We selected fifteen gestures for each robot, including both free-form and task-oriented gestures. We expected that the context or purpose of a gesture might influence the control scheme which participants chose to command it, and so our test set sampled gestures of different types rather than providing a complete assortment of random movements designed to cover the robot’s full workspace. All robot gestures were performed with the base of the robot arm anchored in place, and all motions were performed in the region of space in front of the robot, even if its workspace also extended behind it.

Gestures were presented to participants in the order shown in Table 1, and fell into four categories: The first six gestures are all single-degree-of-freedom motions, and together cover all the degrees of freedom of the robot arm. These were intended to identify DoF-to-DoF mappings, as well as to ensure that the participant was aware of all robot joints. The second category (gestures 7–9) were the first set of task-related gestures: in these motions, the robot reaches for a small spherical target within its workspace. Gestures 10–12 were similar, but required obstacle avoidance as well as target-touching. These gestures were intended to capture differences in control scheme design for cases when intermediate joints must be controlled as well as the end-effector. Gestures 13 and 14 were push-a-block gestures; these gestures required continuous control of the end-effector’s motion, not only at the end of its trajectory. Gesture 15 fell into a different, more semantic category: a wave gesture. As it was designed to be communicative rather than mechanical, this task presented an example of a gesture whose success might not require either precision in either the robot or human operator.

### 4.3 Recruitment

16 participants (10 male, four female, two unreported) between the ages of 18–32 were recruited by email and word-of-mouth in Atlanta, Georgia in the United States. According to the IRB protocol, participants were given two consent forms, one for the study and a secondary, optional video consent form, and compensated with a 12 USD gift card.

Out of our initial 16 participants, 15 were right-handed and only one (P2) was left-handed. Only one participant (P8) had regular prior experience with VR, and six (P3-5, 10–11, 15) regularly handled robots in their personal or professional lives. For self-reported physical activity, 13 participants reported doing physical activity involving gross motor skills at least weekly, and 13 reported doing fine motor skill activities at least weekly. Of note is one participant who self-reported as never doing activities requiring gross motor skills: this was P16, who did the study from a wheelchair.

## 5 Clustering method using trajectory similarity analysis

The goal of data processing is to identify groups of participants with similar enough control scheme preferences that a control scheme can be personalized for the group as a whole. To identify whether such groupings exist and are feasible, we cluster participants according to the similarity of their control motions for each gesture of each robot, where “similarity” is calculated using a distance metric (Absolute Trajectory Error, or ATE) commonly used in SLAM to quantify differences in both position and orientation between two trajectories (Zhang and Scaramuzza, 2018). This allows us to analyze the formed clusters for continuity across robots and gestures and determine each group’s common control scheme rationale.

Before calculating the difference between participant control trajectories, however, we must consider two kinds of natural variation that may be present which may or may not be intentional aspects of the control schemes being demonstrated. The first kind of variation is temporal variation: a participant might lag behind or anticipate the robot’s motion as they demonstrate their control trajectory. When this occurs, a moment-by-moment comparison of trajectory poses might show very large positional differences that are due to small timing mismatches, creating the appearance of dissimilar control trajectories. We can compensate for timing mismatches using Dynamic Time Warping, or DTW, which relabels one trajectory’s timestamps to minimize the distance between all synchronous poses, provided that the order of the timestamps for both trajectories are preserved (Wang et al., 2013; Magdy et al., 2015; Toohey and Duckham, 2015; Tao et al., 2021).

Another kind of between-participant variation that may occur is spatial scaling or translation. Participants with different arm lengths will produce motions at different amplitudes and starting at different heights; these dissimilarities are not necessarily indicative of a difference in control scheme preference. On the other hand, participants who choose to do a motion with their wrist instead of their entire arm will also show differences in spatial scaling and initial pose translation which could be important to their control scheme concept. We can compensate for scaling or translation using Umeyama alignment, a method which scales, rotates, and translates one trajectory on top of another in a way that minimizes the least mean squared error between corresponding trajectory points (Umeyama, 1991).

In the following sections, we will describe a clustering procedure that includes either spatial or temporal pre-alignment in order to identify the benefits and trade-offs of collapsing either type of inter-participant variation. Clustering begins with pre-alignment of pairwise participant trajectories 5.1 and is followed by calculating the ATE between the aligned trajectories to produce a “distance” or dissimilarity metric between participants for each robot gesture. After calculating all pairwise dissimilarity metrics for a given gesture, Agglomerative Hierarchical Clustering is used on the resultant distance matrix to identify groups of participants with distinct control schemes. The validity of the clusters that are based on the reduction in spatial or temporal variance can then be validated with survey data.

### 5.1 Data alignment

The VR software records participant right hand, left-hand, and headset position and orientation in space during each recorded movement and saves them as comma-separate-values (CSV) files. It simultaneously captures the robot’s joint angles and end-effector poses, which are moving through their pre-programmed gesture during the participant’s recording. These CSV files are stored on the headset, to be downloaded for later processing.

Determining the similarity between different control motions first requires that we identify the primary hand used to control that motion. For each gesture, we compared the range of motion of each hand to determine which hand (left, right, or both) had been used for the control movement. The primary hand which performed the motion was the only one used for subsequent steps. Left-handed data was marked for reference, but was used as provided without any additional processing (e.g., mirroring), as no participants chose to mirror the robot’s motion. The hand’s pose data was then centered based on the participant’s initial head position at the start of each demonstration.

**FIGURE 4:**
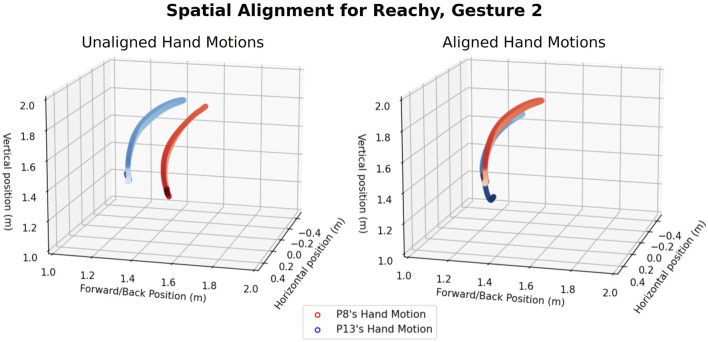
Spatial alignment using Umeyama’s method. The original trajectories are given on the left, with the shade of the color of each trajectory representing the passage time (from light to dark). The trajectories after alignment are shown on the right.

**FIGURE 5:**
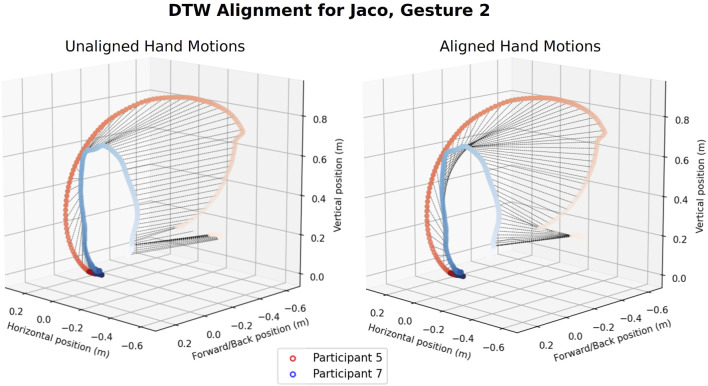
An example of temporal alignment using Dynamic Time Warping. Black lines show the temporal alignment between points on two participant trajectories. Without DTW, P5’s smaller, faster motion would be compared against the early parts of P7’s slower, larger movement. With DTW, time points are aligned such that key parts of the trajectories, such as their inflection points, are compared when calculating the ATE.

We performed two kinds of data alignment prior to computing the distance metrics between pairs of trajectories. Spatial alignment corrects for differences in reference frames, participants with longer or shorter arms, and motions at different scales (e.g., a participant who uses a wrist motion instead of an elbow motion). Spatial alignment between pairs of trajectories was performed using the Umeyama method (Salas et al., 2015; Zhang and Scaramuzza, 2018), provided by the evo package (Grupp, 2017). The effects of spatial alignment can be seen in Figure 4. Temporal alignment is performed using Dynamic Time Warping (DTW) (Salvador and Chan, 2007). Dynamic Time Warping allows us to synchronize motions that may be partially or completely at different velocities, providing a means to correct for participants who lagged behind or anticipated the robot’s movement. The temporal alignment of two trajectories before and after applying DTW can be seen in Figure 5.

Dynamic Time Warping requires a scalar value to compare distances between the two trajectories at each timestep; however our trajectories consist of six dimensional poses. To compute the scalar distance metric, we use a modified version of the Absolute Trajectory Error (ATE) for each pose, which is commonly used in the SLAM/Visual Odometry literature to compare 6-DoF robot trajectories (Zhang and Scaramuzza, 2018). While the original version only makes use of the translation part of the pose for computing the error value, our modified version uses the full pose. We define the error, E, with the following equation:

$$\text{E}=\|\text{log}\left(R_{i}^{-1}SP_{i}\right){\|}_{2}$$

where at time *i*, **R**_*i*_ ∈ SE(3) is the pose of the first participant, **P**_*i*_ ∈ SE(3) is the pose of the second participant, **S** ∈ Sim(3) is a similarity transform computed from the spatial alignment that ensures both poses are in the same coordinate frame, and **log** is the logarithm map which converts the resulting 4 × 4 pose matrix (which is a matrix Lie Group) into the equivalent 6D vector representing the corresponding Lie algebra(which is a vector on the tangent space of the SE(3) manifold) (Chirikjian, 2012). For more details, please refer to Solà et al. (2018).

Once the trajectories are aligned, we can compute the ATE as

$$\text{ATE}=\frac{1}{N}\overset{N}{\underset{i=1}{\sum }}\|\text{log}\left(R_{i}^{-1}SP_{i}\right){\|}_{2}$$

where N is the total number of points in the trajectory. The ATE implicitly computes the Root Mean Square Error (RMSE) between two trajectories, and so is normalized with respect to the trajectory length.

**FIGURE 6:**
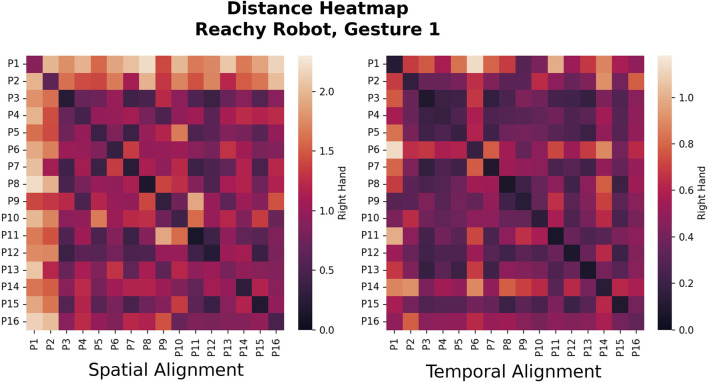
Heat maps showing between-participant similarity for the raise-arm-forward motion of the Reachy (Gesture 1). Each block reveals the Absolute Trajectory Error (ATE) between the pair of participants matching the row/column entry. Low error values are indicative of more similar trajectories after either spatial or temporal variation has been accounted for (spatial and temporal alignment effects are shown separately). The effects of spatial and temporal alignment can be seen on this figure: Participants 1 and two made their command movements quickly, intending to provide an advance command for the robot to follow. Temporal alignment reduced the difference between P1 and two and the other participants, allowing P2 at least to join in the main cluster. On the other hand, P16 did the study from a wheelchair, and performed most of the movements with their wrist instead of their full arm. Spatial alignment compensated for the difference in scaling and allowed P16 to be grouped in with other participants.

**FIGURE 7:**
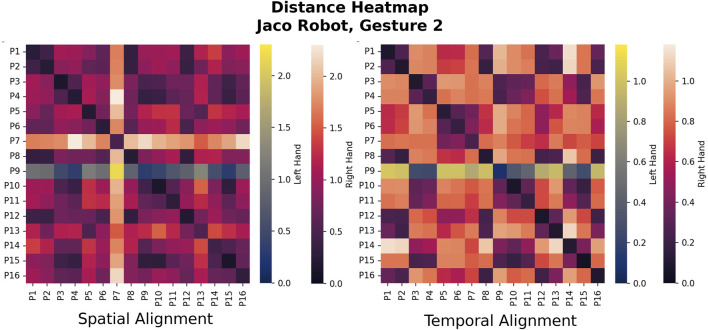
Heatmaps for Gesture two for the Jaco, the “raise arm forward” gesture that is most similar to the Reachy Gesture 1. With the Jaco robot, the control scheme paradigms that correspond with the main clusters are often similar to those of the Reachy, but the participants group differently, as can be visually discerned from these heatmaps. The actual clusters are calculated using Agglomerative Clustering based on a threshold linkage value which was determined by ATE histogram binning.

For each gesture, we compute the ATE for each pairwise set of demonstrations between two participants and average them to compute the overall similarity score between participant trajectories (lower scores mean a smaller differences). Proceeding with all participant combinations, we populate a similarity matrix with the ATEs between all participant pairs for each gesture of each robot. We repeat the process with trajectories that have been aligned temporally instead of spatially. A heatmap of the similarity matrices makes participants with similar/different control trajectories easy to visualize—see Figures 6, 7 for examples.

### 5.2 Clustering

To identify groupings of participants that may have mutually-compatible control scheme preferences, we perform clustering on the control trajectories provided for each gesture. We use the mean Absolute Trajectory Error stored in our similarity matrices, as the ATE captures the difference over the complete trajectory using both the position and the orientation.

**FIGURE 8:**
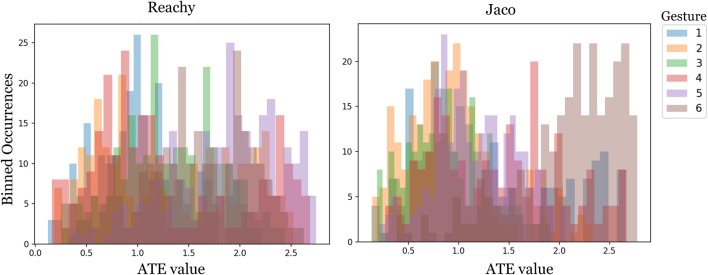
Histogram binning was used to determine the distance threshold for Hierarchical Agglomerative Clustering. Histograms of ATE values for spatial alignment are shown. For spatial alignment, fewer ATE values occur between pairs of trajectories in the 1.2–1.8 ATE range, although the actual least-common ATE varies with gesture. A linkage threshold distance of 1.4 was selected for clustering for both robots for ease of comparison. For spatial alignment, the maximum ATE values were lower, and the distance threshold determined from histogram binning was 0.6.

Since the true underlying number of clusters is unknown *a priori*, we leverage Hierarchical Agglomerative Clustering (Day and Edelsbrunner, 1984) to compute the different clustering levels. We use the gesture’s similarity matrix as the distance matrix and set the linkage criterion to “average”. The distance threshold was selected based on the average bifurcation point of a histogram bar plot of ATE values (see Figure 8).

The similarity matrices shown in Figures 6, 7 allow for visual comparison of the ATE error between trajectories for different users for a single gesture. In this case, the arm-lifting forward motion corresponding to Gesture 1 on the Reachy and Gesture two on the Jaco. For this simple, 1-DoF motion, the clusters between participants are visually distinct, at least for the Reachy robot, though the actual linkage value for distinguishing clusters was determined by the aforementioned histogram binning.

**FIGURE 9:**
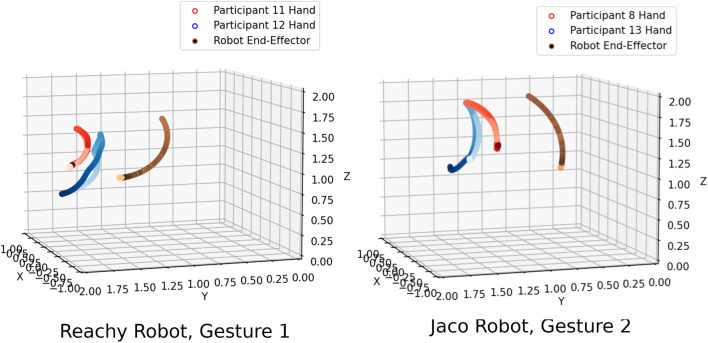
Participants designing motions to control the straight-arm raise for both robots fell almost entirely into two clusters: those that used their hand to precisely follow the robot’s motion (red), and those that did an equivalent arm-raising motion in the natural range of their own arm (blue). Hand trajectories are colored from light to dark to show motion over time.

The participants split up differently for the Jaco, despite the similarity of the gesture; however, Figures 6, 7 show regions of low and high error that still reveal prominent clusters. If participant trajectories are plotted, as in Figure 9, we can see motions that are representative of the hand motions associated with the two main clusters. In this case, the feature that distinguishes the two clusters is the same for both robots: one cluster performed an arm raise that spanned their own full range of motion, while another group raised their arm to match the robot’s position in 3D space. More details on the features that distinguished the clusters that formed for other gestures is presented in Section 6.

## 6 Results

### 6.1 Effect of spatial and temporal alignment on cluster size

Before analyzing the clusters formed for different robots and gestures, we examine the effects of our two different pre-processing alignment techniques. As with the example shown in Figure 9, temporal alignment and spatial alignment compensate for different types of motion scaling and can join users who might otherwise be considered outliers into a larger cluster. The two methods for alignment compensate for variation in trajectory speed via DTW, and scaling/rotation/translation of trajectories via Umeyama spatial alignment. Either of these methods of reducing participant variation will reduce both arbitrary and intentional variations between participants, so we examine them separately in order to identify what cluster types each pre-alignment method hides or reveals.

To see these alignment methods in action we can look at the raw ATE metrics in Figure 6’s similarity heatmap, which show visual differences in group sizes based on the type of alignment which was used. While most of the members of the group were within the threshold for inclusion, P1 and P2 performed their movements quickly, in advance of the robot’s motion and were both considered outliers in the case where temporal alignment was not performed. In the case of P16, who performed the study from a wheelchair, the demonstrated trajectories were performed with the wrist rather than the entire arm. This participant was considered an outlier when temporal alignment was performed, but was joined into a cluster when their motions were scaled by spatial alignment.

**FIGURE 10:**
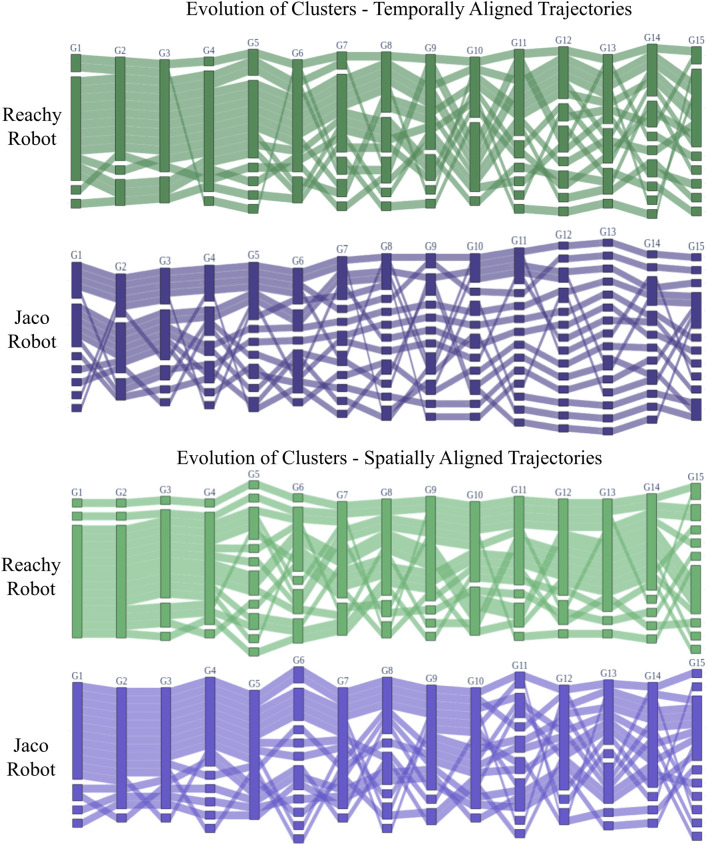
The evolution of control scheme clusters for each robot, with the top plots showing clusters that form for temporally-aligned trajectories and the lower plots showing clusters that form for spatially-aligned trajectories. Participants are represented by rows and gestures by columns, although participant order is rearranged to position users who fall into similar clusters together as much as is feasible. In each gesture column, clusters are shown as groups of participants connected together; clusters are separated from each other by white space. Shaded lines between gestures show how participants rearrange into new clusters for different gestures. Note that the number of non-singleton clusters in a gesture is usually between one and three, depending on the robot embodiment, gesture, and pre-alignment type, and these clusters tend to follow similar paradigms, as shown in Figure 11. However, certain combinations of embodiment, gesture, and alignment have more singletons than multi-participant clusters, which can only be sensibly combined by manual tuning of the cluster threshold value.

In the alluvial diagram showing how clusters formed and evolved across the set of gestures for each robot (Figure 10, temporally-aligned trajectories resulted in more distinct clusters than spatial alignment. This indicates that reducing spatial variation while permitting temporal variation yields larger groups of similar participants. In other words, participants in our study varied more by shape than speed.

Since most participants reported in the post-study survey that their intended control scheme was to map their hand to the robot’s end-effector, this temporal consistency may make sense. On the other hand, many participants reported that they became impatient with the robot’s speed and moved quickly to its end position, the probable cause of many velocity discrepancies. Manual tuning of the threshold clustering parameter allows a designer to more carefully capture the kinds of temporal and spatial variation desirable for their particular focus.

### 6.2 Effect of robot and gesture type on cluster formation

Figure 10 shows that participants split into fewer clusters for the Reachy robot than the Jaco, but that the clusters that formed for the Jaco persisted at least as stably as for the Reachy. Given that the Reachy robot was anthropomorphic and had an obvious one-to-one structural mapping with the user, unlike the Jaco, it makes sense that the Jaco inspired a greater variety of control schemes than did the Reachy.

For both robots, at least one multi-participant cluster formed for most of the gestures, although it persisted most stably in the spatially-aligned case. However, it is worth noting that the individuals who fit into these clusters were different for the two robots. Figures 6, 7 highlight this: for the Reachy, almost all participants fell into the same cluster. However, the Jaco similarity matrix in seven shows that while the Jaco also had one large cluster and some outliers, the breakdown of which participants were in each cluster was different—even though the robot motions the participants were attempting to map to were almost identical. Fortunately, as will be discussed further in Section 6.3, the characteristics of the main clusters were similar for both robots. This suggests that these control scheme paradigms may be worth developing for, even if the paradigm that a particular user will prefer cannot be known *a priori*.

Gesture type and complexity also influenced the number of clusters that formed. Recall that G1-6 are 1-DoF gestures with no environmental interaction, and that succeeding gestures tend to increase in complexity, length, and the amount of environmental interaction until the final, communicative gesture (G15). Figure 10 shows that early gestures tend to have fewer singletons than later gestures, although the small wrist bending gestures in Reachy G5 and Jaco G6 tend to be difficult for spatial alignment to consolidate. Long gestures such as those performed by the Jaco in G11-14 are varied in ways that are particularly difficult for temporal alignment to reduce. The variety in the task-oriented gestures from G7-G14 stems primarily from participants choosing to provide task-relevant motions for the robot to imitate rather than attempting to directly control the robot, which was the more common approach with the first six gestures.

### 6.3 Cluster descriptions based on trajectory data

**FIGURE 11:**
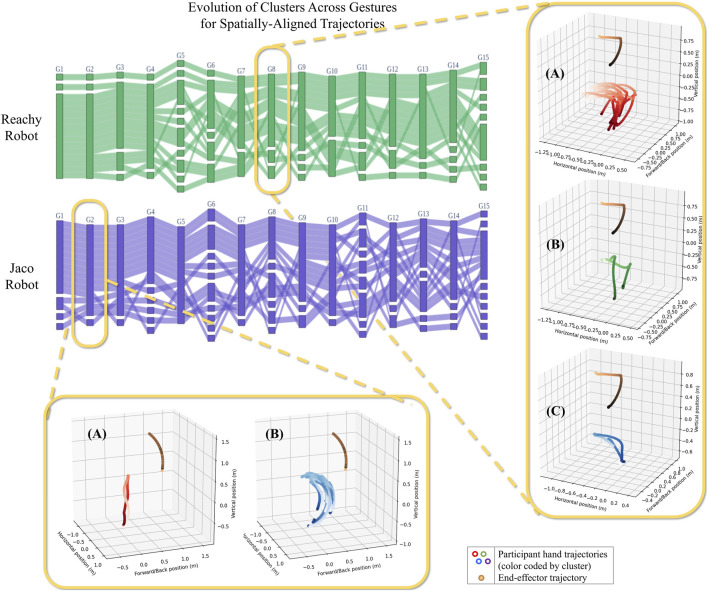
Examples of the clusters that form for different gestures. The end-effector’s motion is shown by orange-to-purple-colored line. For the Reachy (G8) gesture, “touch a ball on the right,” Cluster A was the largest group, with participants who synchronized carefully with the robot in terms of both time and space. In Cluster B, participants still generally followed the robot end-effector’s motion, but their hand trajectories show a looser coupling with the robot in terms of both space and time. For Cluster C, participants moved their hand nearly straight from the start to the end position. In the other callout, for Jaco’s G2 (“Straight arm raise forward”), similar clusters formed, but with different numbers of participants. Participants in Cluster A, one participant again went straight from the start point to the end point. In Cluster B, participants swung their arms to follow the robot’s exact motion.

To understand the types of clusters that may be represented, we use Figure 11. This figure shows example clusters identified after spatial alignment for Gesture eight of the Reachy robot and Gesture two of the Jaco. In the case of Reachy G8 (“reach over a barrier on the right to touch an obstacle”), participants formed three clusters. In the first, participants matched the robot’s end-effector very closely with their hand, matching it in terms of both space and velocity. (Note that the change in color of the hand trajectories from light red to dark red are uniform, representing similar positions at similar points in time. Also note that temporal alignment has not been implemented on this set of gestures to force those gestures to be temporally uniform.) In the second, participants more loosely matched the motion of the robot’s end-effector, with some variation in speed and position. In the third cluster, two participants moved their hand almost directly between the end-effector’s start point and end point, disregarding the intermediate positions.

The clusters that were identified for G2 of the Jaco (“Raise arm in front”) were similar in type to the clusters found in Reachy G8, though this simpler gesture showed fewer overall clusters. The Jaco G2’s Cluster B represents participants who carefully followed the path and timing of the robot’s end-effector, while the participant in Cluster A moved their hand directly from the end-effector’s starting position to its ending position, similar to Cluster C for Reachy G8.

Gestures found after temporal alignment also form groups with similar descriptions, for gestures where clusters can be found. The most common control scheme descriptions across all robots, gestures, and alignment methods involve participants who match the robot end-effector’s motion with their hand in terms of both time and space. A second common cluster maintains the hand-to-end-effector mapping, but loosens it either temporally or spatially—for reasons that are best explained from participant narration and survey data (see Section 6.4). A third, often small, but consistent control scheme is one where the participant represents the start and end points of the robot’s end-effector with their hand, and assumes that the robot will interpolate or do obstacle avoidance as necessary. The Jaco inspired one additional control scheme, adopted and reported by two participants: a two-handed control scheme, where the dominant hand was mapped to the robot’s end-effector and the non-dominant hand mapped to the position of the Jaco’s “first elbow.” This control scheme was not captured by the clustering method described above, which assumed a single-hand control scheme; the fact that this scheme included mapping of the dominant hand to the robot’s end-effector allowed this control scheme to blend in with other single-handed demonstrations. (This highlights an opportunity for future work to extend this method to capture two-handed control schemes. Participants who did not fall into a multi-person cluster for any particular gesture were not considered to be representative of a collective control scheme paradigm within the context of the size of our study.

It is also worth noting that additional control schemes were invented, though they were not used by multiple participants and sometimes did not persist across multiple gestures. However, these unique control schemes—particularly ones that did persist (for instance, the reduced-scale control scheme performed by participant 16, usually visible as the bottom participant row in the temporal alignment plot)—serve as a reminder that additional control schemes may be viable, even if they are not the control schemes that are intuitive to most users.

### 6.4 Cluster descriptions based on participant self-reported control scheme rationales

Trajectory clustering highlighted three predominant control scheme paradigms (shown in Figure 11), but the features distinguishing these clusters were inferred based on visual inspection of the clusters by the first author. To validate and refine these cluster definitions, we compare them with participants’ self-reported explanations of their control scheme rationales. The aim of this analysis is to assess the validity of our cluster definitions, understand participant priorities and concerns, and explore the value of trajectory vs. survey data for personalized control scheme design.

#### 6.4.1 Trajectory-based vs. survey-based control scheme descriptions

The three most prominent clusters identified in Section 6.3 all involved a hand-to-end-effector mapping. In the first, users synchronized between their hand pose and the robot’s end-effector pose in terms of both space and time; in the second, users loosely followed the robot end-effector with their hand but diverged somewhat in terms of speed and/or position; in the third, users only matched their hand to the robot’s end-effector at key points such as the beginning and the end. This hand-to-end-effector mapping is corroborated by participant descriptions: in post-study surveys, all participants mentioned incorporating a hand-to-end-effector mapping in their control scheme concepts for both robots. In the case of the Reachy, participants described their control scheme as “mimicking the robot” in its entirety (arm-to-arm mapping was both demonstrated and reported by 100% of participants, as was noted by the first author, based on participant arm positioning for barrier-avoidance gestures). For the Jaco, users were much more likely to focus only on the end-effector (demonstrated by 100% of participants based on arm position during barrier-avoidance gestures; mentioned by 50%). Still, 38% of users stated that they tried to match the entire Jaco arm for gestures 1-6, while motions were simple enough that this was still possible. On the flip side, 13% of users noticed discrepancies between their limb lengths and the Reachy’s arm length and had to decide how to scale their motion so that it would still “match.”

While the hand-to-end-effector mapping was nearly universal, the majority of the stable control scheme clusters showed that temporal and spatial synchrony between the two was not. Many participants were aware of this and reported their intentional change in speed or range of motion in their control scheme descriptions. The percentage of users who demonstrated temporal or spatial deviations matches the clusters observed through trajectory similarity analysis, validating that trajectory-based clustering can identify conceptually different control schemes. The rationales behind these adjustments were provided by participants verbally during the test, as well as in the post-test survey, and are shown in Table 2. It is worth noting that not all participants mentioned their spatial or temporal adjustments when explaining their control schemes. Some participants took spatial or temporal scaling for granted, although participants were much more likely to comment on deviations from the robot’s speed and/or motion than on their synchrony with it.

**TABLE 2:** Users described the ways they diverged from exactly matching the robot’s end-effector, spatially and temporally.

User behavior	Explanation(s)	Percentage of users reporting or demonstrating this behavior
Synchronized spatially and temporally with the robot		Reachy
		13% reported
		38% demonstrated
		Jaco
		6% reported
		38% demonstrated
Temporal Variations		
Went faster than the robot	Were impatient with the robot’s speed	Reachy
		31.25% reported
		38% demonstrated
	Intended to command the robot in advance, not in real time	Jaco
		6% reported
		13% demonstrated
Spatial variations		
Reduced scale or range of motion	Fatigue/ergonomic reasons	Reachy
		13% reported and demonstrated
		(1 additional person suggested it without doing it)
		Jaco
		19% reported
		25% demonstrated
	Skipped uncomfortable or extraneous robot motions	Reachy
		19% reported
		25% demonstrated
		Jaco
		38% reported
		44% demonstrated(Jaco)
	Exaggerated motion to convey intention	Reachy
		31% reported
		38% demonstrated
		Jaco
		6% reported and demonstrated
	Focused on task; let the robot figure out the rest	Reachy
		19% reported and demonstrated
		Jaco
		31% reported
		50% demonstrated

Participants were also more likely to comment on spatial or temporal variations if their control scheme had changed to include or exclude them over time. Temporal changes included shifts away from and towards synchronizing with the robot. Users who departed from temporal synchrony sped up over time due to impatience; users who did the reverse began with fast, possibly semantic motions, and then synchronized with the robot’s end-effector as its motions became more complex. Participants who changed their spatial scaling over time generally moved towards a smaller range of motion: they became physically tired from trying to match the robot’s full range, and scaled it down as the study progressed. One exception to this is in the case where a participant exaggerated a movement to provide additional context: Reachy’s Gesture 10 (“Reach underneath a barrier to touch a target”) required the same elbow-bend motion as in Gesture 4, but some participants bent over or enlarged their motion in order to emphasize the need for the robot to go around the barrier.

The fact that trajectory analysis revealed nuances in participants’ control schemes that were not always or fully captured by survey responses suggests that both kinds of analysis may be required to fully understand participant preferences on their control scheme paradigms. Surveys allow participants to describe the rationales behind the mappings they intend, which trajectory analysis does not provide; on the other hand, trajectory analysis can disambiguate between participants who describe their control paradigms similarly, allowing us to uncover aspects of their control schemes that could be relevant to control scheme and interface designers.

#### 6.4.2 Participant commentary on their control scheme choices

In post-study surveys, all users mentioned that for most (if not all) of the study, their control scheme concept had been based on a mapping between one of their hands and the robot’s end-effector. This was true even for the Jaco robot with its non-anthropomorphic joint configuration, and even for gestures where control of intermediate joints was important. Participants reported that without the ability to iterate through control scheme options or view all gestures simultaneously, they began with this end-effector-focused control scheme as their default, and most of them maintained it throughout the study for the sake of consistency even as they became aware of its limitations. When presented with obstacle-avoidance tasks, these consistency-focused participants decided to assume that the robot had the intelligence to do whatever obstacle-avoidance and inverse-kinematics calculations were required, rather than adopt a new control scheme that more completely specified the robot’s motion:

*P4* – “Originally I moved to mimic all the motions of the Jaco arm, i.e. mapping its shoulder joint to mine, elbow, etc., but once it started to do motions that I am not capable of, I would think of just moving my hand as the end-effector and imagine it is dealing with all the joint movements itself, which was especially the case during the demo of it swinging its arm over a glass wall to get to the ball just behind it. So, in the end I just mimicked the end-effector of the arm with my hand and ignored the shoulder and elbow movements for complicated actions.”

*P5* – “I just simplified my gesture….If the robot needed to do all of those other movements to orient itself the appropriate way, I would expect it to do those on its own rather than me specifically having to change my gestures to accommodate for the robot’s physical limitations.”

In the post-study survey, seven of the sixteen participants expressed concerns about the limitations of their final control scheme. Although they had the ability to fully specify the robot’s configuration with their own arm’s 6 DoF (whether the 6-DOF of their hand’s position/orientation or the 6-DoF associated with the joint motions available between their shoulder and wrist), they said that they had not provided complete directives to the robot with their choice of motions and asked if the robot could be expected to be intelligent enough to fill in the missing pieces.

Individuals also expressed concerns about ergonomics and fatigue. According to notes taken by the first author during the studies, at least five participants chose to match the robots’ motions exactly, going to the limits of their own reach despite mid- or post-study complaints about operating at the limits of their range of motion. Five others were noted for adjusting their posture at the ends of trajectories to avoid discomfort, while only two intentionally scaled down their control motions at any point during the study according to survey responses. Of these two, the only one who consistently operated at a reduced scale had a disability that made them unusually susceptible to muscle fatigue. Most other participants expressed concerns about the long-term ergonomics and fatigue effects associated with a hand-to-end-effector mapping as the operating control scheme, but for cognitive simplicity and the sake of consistency, they chose and maintained that mapping.

*P6* – “I’m just trying to mimic it because that’s easier for me to remember. My brother is more of a gamer person, so he might choose more abbreviated motions to represent the whole thing. But for me, this is easier.”

*P12* – “Because I’m mimicking his movements–I’m thinking, ‘if this robot was my arm, how would I move it?’ But in real life, I’d probably want a smaller range of my own motion to correspond to the robot’s motion. It’s just that I can’t see my own movements, so I exaggerate.”

*P16* – “Because of my disability and muscle fatigue limitations, I became aware of the strain on my arm very quickly. Even though it made sense to do full arm motions, I tried to reduce effort by doing fine wrist motions to control the robot’s gross arm motions (it seemed like 90% of what the robot was doing), and I further reduced muscle strain by anchoring my elbow on my arm rest when possible.”

Five participants mentioned a different concern: that their control scheme was limited to a human range of motion and would not be adequate for controlling the robot in its full workspace. Their reasoning for retaining a hand-to-end-effector mapping despite this limitation was based on 1) their own urge for consistency (a priority expressed by 25% of participants, although one participant took the opposite view), 2) they believed that a more sophisticated control scheme could only be invented iteratively, with knowledge of the robot’s full capabilities, 3) mental convenience, as exemplified by the following anecdotes:

*P6* – “I want a motion that helps me keep track of where all the joints are going to go. Maybe the robot doesn’t need that, but I want to keep track so I don’t make it hit anything.”

*P8* – “There’s a difference between what’s conceptually and physically easy.”

*P12* – “This is more about remembering—it’s a memory test.”

#### 6.4.3 Alternative control scheme paradigms

Participant 8’s quote—“There’s a difference between what’s conceptually and physically easy”—is crucial to note for future studies on user-centered control scheme design. Participants universally defaulted to mapping their hands to the robot’s end-effector. Though there were variations in how this mapping was interpreted (did it require strict temporal synchrony? Exact spatial correspondence? Did it only apply at key points of the motion?), every participant recognized and, at some point, used this mapping. For designers attempting to create intuitive control schemes for a broad swath of users, this would seem to be an ideal control scheme, and indeed, it is the control scheme identified by both our trajectory analysis and survey results. However, participants’ concerns about the limitations of their control schemes are valid, and indicate that the most intuitive control scheme may not be one that is functional in the long term. As P8 identified, there is a difference between what is cognitively and what is physically easy, and on a first pass, participants were optimizing for the former.

Further research is needed to determine if longer-term studies with more iterative design methods lead to more physically-optimal control scheme paradigms, and to determine whether such control schemes are also clusterable. What we can tell from this study is that 38% of participants identified at least one of their control scheme’s limitations, and that these same participants were able to brainstorm alternative, non-pose-to-pose and hand-to-end-effector mappings. Some of these schemes were demonstrated (as outliers in the alluvial plot), and many were merely described as participants brainstormed control motion choices but ultimately erred on the side of cognitive simplicity and consistency across gestures.

Alternative control scheme suggestions include:

• Two-handed control of Jaco robot to increase the number of DOF available for control inputs: one hand to operate the end-effector, and the other to operate the “first elbow.” (P10, P11) (See the second participant in Figure 2 for an example.)
• Semantic hand gestures used as a kind of sign-language to tell the robot which of a pre-defined set of gestures to perform (P1, P2)
• (Suggested but not demonstrated): A control scheme involving buttons, to tag individual objects based on the type of interaction expected (i.e., “avoid,” “go through,” “grab”). The robot would then be allowed to figure out its own path. (P9)
• One arm bent or a button pressed to indicate which joint is being controlled by the wrist of the opposite hand (P10)
• To control the robot’s full wrist rotation: a spiral motion with the other hand (P11); a wrist “flick,” with the speed intended to indicate the distance to be rotated (P13); a screwing motion for more precise rotation control (P13)

For final determination of a functional control scheme, participants also requested an iterative process where they could test and refine their control schemes with feedback from the robot. Participants 6, 8, 9, 11, and 12 reported that the lack of foreknowledge of the robot’s movements and/or their inability to iteratively refine their control scheme constrained them to the hand-to-end-effector mapping that they maintained.

## 7 Conclusion

In this paper, we utilized trajectory similarity analysis to analyze the control motions contributed by 16 users for two robots, one anthropomorphic and the other non-anthropomorphic. We discovered that participants demonstrated both individuality and commonalities, split into two or three clusters based on the similarity of their control motions, supporting the hypothesis (from RQ1) that neither a single universal control scheme nor completely individual personalization is necessary for control scheme design.

Addressing 2, we discovered that clusters were similar for both robots in their underlying rationale, and did not appear to change based on the type of gesture that the users were being asked to control. More complicated gestures and less anthropomorphic robots did tend to produce more additional, outlier control motions than the alternatives.

There was a unifying concept underlying the control schemes for all the predominant clusters: a pose-to-pose mapping between one of the user’s hands and the end-effector of the robot. Different interpretations of the nature of this mapping led to the three main clusters observed. For one cluster, this mapping required exact spatial and temporal synchrony between the hand and end-effector. For another, strict temporal synchrony was not required, and slight adjustments of the spatial mapping might also be permitted (e.g., moving the hand in a range relevant to the user’s frame of reference rather than the robot’s). For the last small but consistent cluster, the control scheme only required matching the hand pose to the end-effector pose at key points, such as the start and the end.

Importantly, and answering 3, trajectory similarity analysis was able to differentiate between these groups of users, even when they described their control scheme rationale simply or similarly. The key features that visually distinguish between the identified clusters can be found in participants’ descriptions of their control scheme rationales, validating that objective clustering and trajectory similarity analysis is able to identify meaningful distinctions between control scheme paradigms. Combining trajectory analysis with participant survey data allows designers to capture important kinematic information about user control scheme preferences and also allows them to understand the rationale behind the observed differences.

Some of these differences in control scheme preference may be irrelevant to a designer’s goals, and they may make a conscious decision to ignore them. Since participants were largely consistent about mapping their hand to the robot’s end-effector, a participant can likely accommodate a designer’s choice as to the timing of the command or the scale of the motion required to teleoperate the robot. This statement does not imply that all timing/scaling choices are equally useful. For instance, participants expressed concerns about fatigue and ergonomics over the long-term when using large control motions, and prior research on time delays in teleoperation suggest that best-case performance occurs when latency is as close to zero as possible (Lee et al., 2022).

Considering timing and scaling from the user perspective, participants often made explicit note of their deviations from the robot’s speed or movement as though assuming that the default control scheme should be synchronous with the robot in time and space. A control scheme that follows the robot’s movement closely spatially and temporally is more likely to be intuited by a new user than one that is scaled or time-shifted toward a specific prior user’s preferences—but since the largest clusters of participants were time- and space-synchronous, choosing a clustering threshold value that incorporates asynchronous participants into the main cluster will likely still tend toward a synchronous control scheme.

Several users noted limitations of the control schemes they had selected. Although the identified clusters seemed to draw upon a nearly universal control scheme concept, making them highly intuitive, participants noticed that a hand-to-end-effector mapping placed restrictions on the robot’s range of motion and required the user to execute large, fatiguing, and sometimes uncomfortable motions. One participant said it succinctly: “there’s a difference between what’s conceptually and physically easy,” and participants’ initial choice of control scheme preferred the former. The control schemes that our participants invented on a first pass were cognitively intuitive, but probably ill-suited for long-term use or full functionality. Participants suggested and/or attempted alternative control schemes as a first attempt at resolving these concerns: some utilized both hands to control a single arm, while others attempted to communicate the type of gesture desired through sign language (or requested a button interface) and expected the robot to perform its own path-planning. Still others wanted a button or gesture that allowed control mappings to change during use, or invoked specific, physics-inspired motions to command joints moving beyond their personal range of motion. Further research is necessary to identify if iterative control scheme design resolves some of the limitations of the demonstrated control schemes. Future work can also investigate whether participants continue to fall into clusters with similar preferences if the physical constraints, rather than the cognitive ones, are prioritized.

Additional research is also warranted in several areas which were simplified for the scope of this study. One simplification was the restriction of user inputs to 6 DOF hand poses rather than unconstrained hand and finger motion. This reduced the complexity of the gestures that participants could demonstrate down to the theoretical minimum number of degrees of freedom which could be used to fully control the robot arms. While there are computational advantages to matching the DOF of the inputs with the outputs, this constraint likely influenced the control scheme paradigms that participants produced. Future experiments that record unconstrained hand motion could be used to determine 1) the effect of increased freedom in control inputs on the clustering of users’ control scheme preferences, and 2) what particular movements or combinations of movements [usually called “synergies” (Vinjamuri et al., 2010; Scano et al., 2018)] would be most useful as control inputs if 6DOF controls are desired.

An additional opportunity for future research comes from the set of robots we studied. Participants consistently conceptualized a mapping between their hand and a robot end-effector, which was a convenient control scheme for two arm-like robots. It would be valuable to conduct another study with other kinds of robots to understand how control schemes differ for other robot shapes, as well as the impact shape may have on the feasibility of groupwise personalization.

Finally, we note that participants did not have the chance to use the control schemes they demonstrated, and there is opportunity for further research on the functionality of those schemes. Allowing participants to test out their control schemes would also permit research on how participant preferences evolve if they are allowed to develop their mappings over multiple iterations, as several participants suggested. The single-pass data collection design of our study allowed us to capture user preferences prior to any inadvertent training: in normal operation, the robot’s real-time responses provide feedback to the user, who may adapt their control motion during the gesture to encourage the robot to perform the desired motion. This was important for understanding commonalities in user preferences; however, without real-time feedback (and with goal gestures that were not strongly time-sensitive), the specificity of participants’ command motions may have been correspondingly loose. Our research suggests that functional control schemes can be developed using sensibly-combined data from multiple user demonstrations, but to accomplish this will likely require multiple iterations of data collection and testing as well as sophisticated processes for merging data from multiple user demonstrations.

## Data Availability

The datasets presented in this article are not readily available because data access is restricted to the participating authors on the study by the Georgia Tech Institutional Review Board. Requests to access the datasets should be directed to jmolnar@gatech.edu.
